# Disseminated phaeohyphomycosis in a dog

**DOI:** 10.1016/j.mmcr.2017.02.003

**Published:** 2017-02-24

**Authors:** Lana S. Rothenburg, Timothy A. Snider, Allison Wilson, Anthony W. Confer, Akhilesh Ramachandran, Rinosh Mani, Theresa Rizzi, Laura Nafe

**Affiliations:** a Oklahoma State University, Department of Veterinary Clinical Sciences, 2065 W Farm Road, Stillwater, OK 74078, USA; b Oklahoma State University, Department of Pathobiology, 250 McElroy Hall, Stillwater, OK 74078, USA; c Oklahoma Animal Disease Diagnostic Lab, 1950 W Farm Road, Stillwater, OK 74078, USA

**Keywords:** Phaeohyphomycosis, Bipolaris spicifera, Disseminated, Canine, Cyclosporine

## Abstract

Phaeohyphomycosis is a rare but emerging disease caused by dematiaceous fungi. Here we describe the case of an immunosuppressed dog with disseminated phaeohyphomycosis secondary to *Bipolaris spicifera* infection. Regionally extensive infiltration of the paw pads, skin, myocardium, liver, renal interstitium and diaphragm was identified on histopathology. *Candida glabrata* and *Fusarium oxysporum* were also cultured from multiple sites post-mortem. The dog was treated with fluconazole, itraconazole, terbinafine and liposomal amphotericin B, but was euthanized due to its poor prognosis after 12 days of therapy.

## 1 Introduction

Phaeohyphomycosis is a term used to describe infection caused by dematiaceous (pigmented) fungi belonging to over 60 genera within the orders *Pleosporales, Ochroconiales*, *Chaetothyriales, Capnodiales, Dothideales, Botryosphaeriales, Microascales, Sordariales, Calosphaeriales* and *Ophiostomatales* [1]. These are typically non-pathogenic soil saprophytes found ubiquitously around the world; however, they can be responsible for life-threatening opportunistic infections in an immunocompromised host [1]. Phaeohyphomycosis has been documented in invertebrates, cold-blooded vertebrates, birds and numerous mammalian species, including ruminants, horses, dogs, cats and humans. A wide variety of clinical syndromes associated with phaeohyphomycosis has been identified, including localized or generalized cutaneous and subcutaneous infections, fungal keratitis, pneumonia or localized pulmonary infections, cerebral abscesses or encephalitis, and disseminated infections [2]. Here we report on a case of disseminated phaeohyphomycosis with concurrent candidiasis and *Fusarium* sp. infection in an immunocompromised dog. This is the third known case of phaeohyphomycosis secondary to *Bipolaris* sp. infection in the dog, and the first to report treatment outcomes.

## 2 Case

A 6-year old spayed female Miniature Australian Shepherd was presented for evaluation of several ulcerative and inflammatory lesions on the pads of all four paws (day 0, date of first symptom ultimately attributed to phaeohyphomycosis). On physical exam, a deep fissure was present in the left metacarpal pad, which was associated with a small amount of serosanguineous exudate. Several digital pads on the remaining three feet had small areas of purple to black discoloration on their surface. The remainder of the physical exam was unremarkable.

The dog was diagnosed with immune-mediated thrombocytopenia (IMTP) three weeks prior, and was hospitalized for supportive therapy relating to severe gastrointestinal hemorrhage secondary to her IMTP (days −25 to −15). She required multiple blood product transfusions, and vincristine (0.015 mg/kg IV) was administered once on day −24 to stimulate platelet release from the bone marrow. Current medications included prednisone (1.1 mg/kg PO in AM, 0.74 mg/kg PO in PM), cyclosporine (5.5 mg/kg PO q24hr), doxycycline (3.7 mg/kg PO q12hr), melatonin (0.22 mg/kg PO q24hr), omeprazole (0.74 mg/kg PO q12hr), and mirtazapine (0.28 mg/kg PO q24hr).

The dog was hematologically stable by day 0, with a platelet count of 252,000/uL (Reference Interval, RI: 170,000/μL-400,000/μL) and a hematocrit of 36% (RI: 36–60%). However, a chemistry profile revealed newly elevated liver enzymes: AST 76 IU/L (RI: 15–66), ALT 575 IU/L (RI: 12–118), ALP 448 IU/L (RI: 5–131) and hyperbilirubinemia (0.7 mg/dL, RI: 0.1–0.3). Cytology of an impression smear from the open left metacarpal pad yielded suppurative inflammation with predominantly degenerate neutrophils and rare macrophages in a proteinaceous eosinophilic background. No infectious organisms were appreciated, but a sterile culturette of the wound was submitted for aerobic bacterial culture (Agar Gel Amies with Charcoal, COPAN Diagnostics Inc, Murrieta, CA). The dog was presumptively started on treatment with niacinamide (37.0 mg/kg PO q8hr) and pentoxifylline (22.2 mg/kg PO q12hr) for immune-mediated vasculitis until further diagnostic tests could be performed.

Culture of the wound swab on 5% sheep blood agar did not yield bacterial growth; however, on day +2, growth of small numbers of fungal colonies was observed. Fungal colonies were subcultured on Sabouraud's dextrose agar and identified as *Bipolaris* sp. based on morphologic characteristics. Cyclosporine was discontinued at that time, but prednisone was continued with the dose unchanged. Treatment with fluconazole (8.7 mg/kg PO q12hr) and tramadol (4.3 mg/kg PO q8-12hr) was also initiated on day +2.

**Fig. 1:**
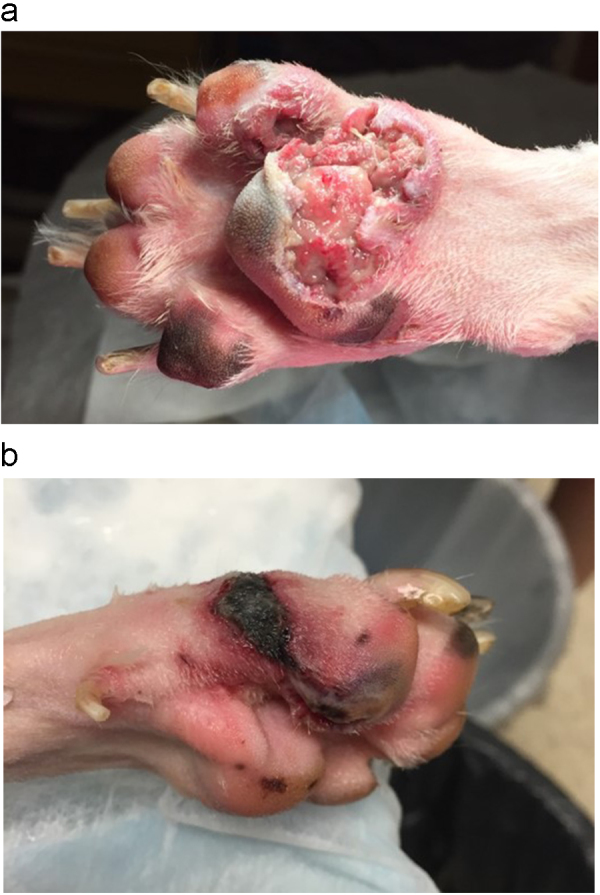
Paw pad lesions observed on day +3, just prior to biopsy. A) Full-thickness ulceration and sloughing of the left metacarpal pad and fifth digital pad of the left forelimb. B) Eschar extending along the plantaromedial aspect of the left hind paw.

Punch biopsies of the right metacarpal pad and fifth digital pad of the right rear limb were performed on day +3. By that time, all the paw pad lesions had progressed. The dermal tissue of the left metacarpal pad had sloughed, revealing the underlying adipose tissue. This sloughing extended through the caudal aspect of the fifth digital pad of the left forelimb (Fig. 1A). A similar necrotic lesion was noted on the third digital pad of the right forelimb. The right metacarpal pad was erythematous and edematous, but had not yet sloughed when biopsies were performed at this site. In addition, pitting subcutaneous edema extended proximally up the right forelimb to the level of the mid-antebrachium. A 2-cm long eschar was present along the plantaromedial aspect of the left hind paw (Fig. 1B). Lastly, erythema and edema were also appreciated on the fifth digital pad of the right hind limb, and punch biopsies were obtained from this site.

**Fig. 2:**
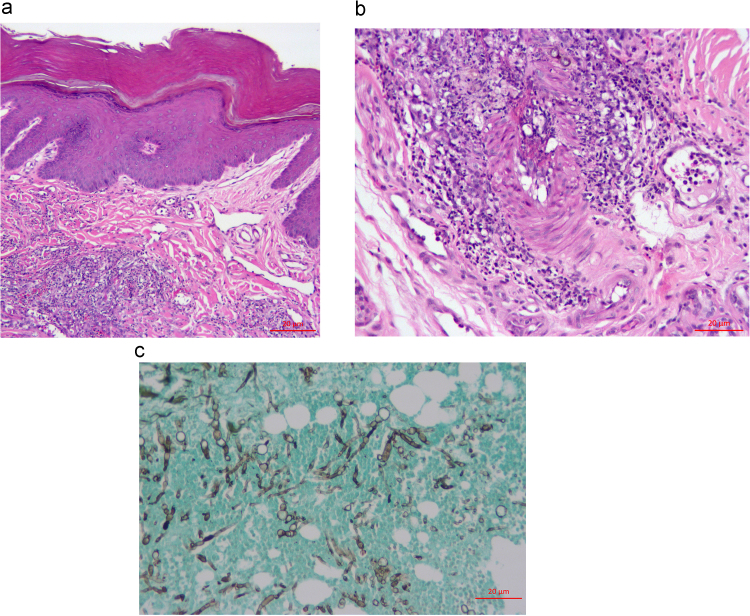
Biopsy findings on day +3 A) Superficial skin from the foot. There is marked hyperkeratosis and acanthosis with a moderate perivascular to interstitial mixed inflammatory infiltrate within the outer dermis. Hematoxylin and eosin stain. Bar=20 µm. B) Superficial skin from the foot, further magnification. Mixed perivascular to interstitial inflammation is composed of many neutrophils, fewer macrophages, eosinophils, and lymphocytes and plasma cells, and occasional gray-brown to negative profiles of fungal elements are seen. The wall of an artery is asymmetrically infiltrated with inflammatory cells. Hematoxylin and eosin stain. Bar=20 µm. C) Superficial skin from the foot. Myriad gray (silver) staining fungal elements, including branching, irregularly septate, hyphae with non-parallel walls and with terminal bulbous morphology are seen infiltrating host tissue elements staining green. Grocott's methenamine silver (GMS). Bar=20 µm. (For interpretation of the references to color in this figure legend, the reader is referred to the web version of this article).

Histopathology of the right metacarpal pad revealed full-thickness epidermal ulceration and infiltration of the dermis with lymphocytes and plasma cells (Fig. 2A). The dermal vessels were frequently thrombosed with vascular necrotic debris and neutrophilic inflammation. The deep adipose tissue was focally necrotic with moderate neutrophilic and histiocytic inflammation (Fig. 2B). No infectious agents were initially identified on H&E staining, and an immune-mediated vasculitis was suspected considering the dog's history of immune-mediated disease. However, fungal culture again yielded *Bipolaris* sp. Subsequent Gomori's Methenamine Silver (GMS) staining demonstrated many fungal hyphae and yeast-like bodies within dermis, epidermis and vessel walls (Fig. 2C).

Antifungal susceptibility testing performed on fungal isolates cultured from the biopsy sample (University of Texas Health Science Center, San Antonio, TX) yielded the following minimum inhibitory concentrations: fluconazole 16 mcg/mL, itraconazole 0.5 mcg/mL and posaconazole 0.25 mcg/mL (≤1 mcg/mL suggestive of potential susceptibility). A 24-hr trough, whole blood cyclosporine concentration was significantly above the therapeutic range (1731.4 ng/mL; RI 400–600 ng/mL; Clinical Pharmacology Laboratory, Auburn University, Auburn, AL).

The dog was initially treated as an outpatient. Soft padded bandages were placed on all four paws after the biopsies were performed, and the bandages were changed on days +4 and +5. Necrotic tissue was debrided when indicated, and miconazole cream (2%) was applied to the contact layer of the bandages. The dog became progressively more anorexic, lethargic and lame in the days subsequent to the biopsy. Her dose of prednisone was decreased to 0.87 mg/kg PO q12hr on day +4. Due to persistent anorexia and unwillingness to take oral medications, the dog was hospitalized on day +7 for ongoing wound care, pain management and supportive therapy. Bandages were changed every other day. Enrofloxacin (9.3 mg/kg PO q24hr) was initiated on day +7 given the growth of a susceptible *E. coli* infection on aerobic wound culture. The dose of prednisone was further reduced to 0.87 mg/kg PO q24hr on day +7. On day +8, fluconazole was discontinued and replaced by itraconazole (9.1 mg/kg PO q24hr) and terbinafine (28.4 mg/kg PO q12hr). Low numbers of hyphal elements surrounded by inflammatory cells were identified on impression smears obtained from the paw pad lesions on the left and right forelimbs on the day +9 bandage change. The hyphae were described as lightly basophilic staining, branching, and septate. They occasionally had oval to large, round conidia-like terminal elements.

An irregularly irregular arrhythmia was ausculted on day +9, and continuous ECG monitoring confirmed frequent ventricular ectopic beats occurring in singles and couplets, with occasional runs of a slow, non-sustained idioventricular rhythm. Cardiac troponin I (cTnI) was markedly increased (5.36 ng/mL, RI: <0.1 ng/mL). An echocardiogram revealed suspected pseudohypertrophy of the left and right ventricles secondary to hypovolemia, with no evidence of vegetative valvular lesions. The myocardium appeared normal in echotexture, and contractility was normal with a fractional shortening of 35%. The dog's arrhythmia progressed on day +11 to include short runs of ventricular tachycardia with occasional R-on-T morphology. Lidocaine (2 mg/kg IV once followed by a CRI at 60 mcg/kg/min) was initiated and provided adequate control of the arrhythmia.

Liposomal amphotericin B (0.7 mg/kg IV over 2hr) was administered on day +11, with plans to continue treatment every 48 hr. However, the owners elected humane euthanasia on day +12 due to the dog's continued clinical deterioration and poor long term prognosis.

**Fig. 3:**
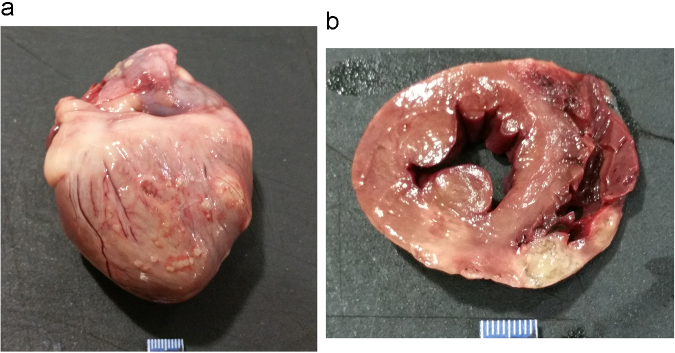
Gross findings of the heart at necropsy. A) Entire. Heart is positioned with right ventricle visualized, along with much of the right atrium. Disseminated to confluent 1–4 mm white nodules affect right ventricular epicardium, along with two larger 8–10 mm foci at the right margin. A minimum of two discrete 2–5 mm white foci are visible upon the right atrial epicardium. B) Cross section. Two pale tan to green foci of transmural necrosis and inflammation are seen. The larger, 11×7 mm, is at the bottom of the image, near the junction of the right ventricular free wall and the interventricular septum. The smaller, 7×6 mm, is to the right of the larger one, with the two separated by 4 mm of less affected right ventricular free wall. Additional variably sized to confluent foci of hemorrhage and necrosis affect the remainder of the right ventricular free wall.

On gross necropsy, deep, regionally extensive ulceration of multiple paw pads was observed. Two circumferential, irregularly circular, 4 cm diameter ringed areas, brown in color with central clearing, were noted in the thoracic skin. Focal bruising was noted within the left mid-abdomen and right caudal thigh. Foci of necrosis were appreciated on the surfaces of the left and right atria, along with several nearly transmural foci of necrosis in the right ventricle, interventricular septum and left ventricle near a site of superficial coronary artery branching (Fig. 3). Both kidneys were swollen on cut section. The cortex of the left kidney contained a circumscribed focus of necrosis. The liver had a few small, irregular foci of discoloration (measuring up to 3×5 mm).

**Fig. 4:**
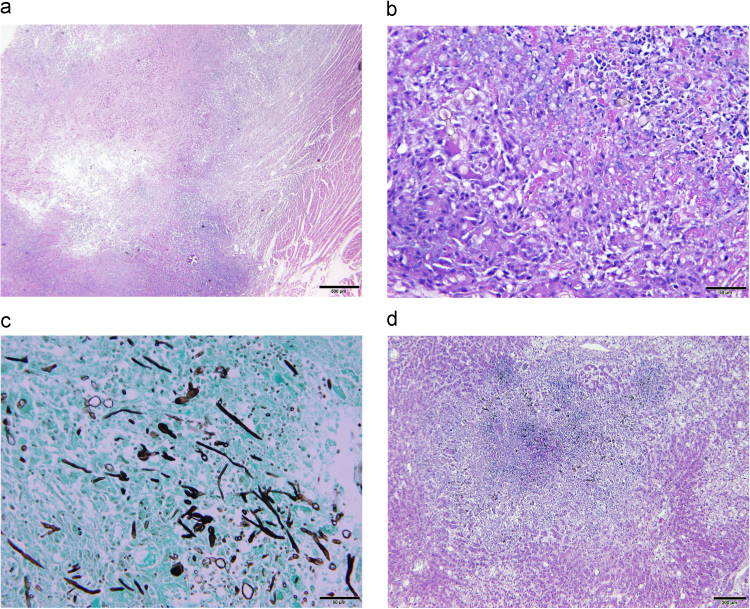
Histopathology findings at necropsy. A) Heart. Central myocardial necrosis is surrounded by pyogranulomatous inflammation. Hematoxylin and eosin. Bar=500 µm. B) Heart. Cardiac myocytes are largely replaced by pyogranulomatous inflammation accompanied by patchy zones of necrosis. Additionally, occasional gray-brown to negative profiles of fungal elements are seen. C) Heart. Myriad gray (silver) staining fungal elements, including branching, irregularly septate, hyphae with non-parallel walls and with terminal bulbous morphology are seen infiltrating host tissue elements staining green. Additionally, other fungal elements are thinner in hyphal profile and are more regular in their branching arrangement. Grocott's methenamine silver (GMS). Bar =50 µm. D) Liver. A random focus of necrosis with surrounding pyogranulomatous inflammation spans portions of multiple hepatic lobules. Gray-brown fungal elements are frequently seen in the pyogranulomatous infiltrate. Hematoxylin and eosin. Bar =200 µm. (For interpretation of the references to color in this figure legend, the reader is referred to the web version of this article).

On microscopic examination, pyogranulomatous inflammation was identified in the myocardium and epicardium, renal interstitium, liver, inguinal and thoracic skin, paw pads and diaphragm (Fig. 4). The inflammation was composed predominantly of epithelioid macrophages, occasional multinucleated giant cells, neutrophils, and lesser numbers of lymphocytes and plasma cells. Larger foci of pyogranulomatous inflammation had significant central regions of necrosis. The inflammation was loosely cellular and usually clustered around small numbers to large clusters of brown pigmented, branching hyphal elements and cells. The hyphae were frequently septate, with crudely parallel walls that swelled slightly mid-segment and had terminal narrowings at points of septation. Fungal hyphal walls were 4–6 µm in diameter and were of variable length. Occasionally, separate ellipsoid fungal structures 3–4 µm in diameter and 4–8 µm in length were identified and believed to be compatible with *Candida* pseudohyphae. Though usually recognizable on H&E stained sections, they were highlighted with GMS staining. The bone marrow had a paucity of megakaryocytes. No histopathologic abnormalities were identified in the brain, pancreas, intestines, stomach, urinary bladder, endocrine glands, lungs and spleen.

Fungal organisms were detected by culture from the heart, paw and kidney. Based on microscopic morphology, these were identified as *Bipolaris* sp. (heart and paw) and *Fusarium* sp. (kidney). Partial 28 S ribosomal gene sequencing (Eurofins Genomics, Louisville, KY) of the fungal isolate initially identified as *Bipolaris* sp. revealed 100% sequence homology with *Curvularia spicifera* (GenBank: KU729203) while the *Fusarium* sp. had 100*%* sequence homology to *Fusarium oxysporum* (GenBank: KT236325). Both these sequences have been deposited in the GenBank database (GenBank KX578213 and KX578214). Recent reports have suggested that based on phylogenetic analysis, *Bipolaris spicifera* may be renamed as *Curvularia spicifera* [3]. Lastly, yeast colonies were cultured from the liver, kidney, brain and heart, and were identified as *Candida glabrata* by MALDI-TOF mass spectrometry (Bruker Daltonics, Billerica, MA).

## 3 Discussion

Disseminated phaeohyphomycosis secondary to *Bipolaris* spp. infection has been previously reported in two other dogs. The first case, also from Oklahoma, was presumed to be secondary to immunosuppression with corticosteroids [4]. The dog died suddenly, and subcutaneous draining tracts were noted on necropsy in both hind limbs, with similar ulceration and necrosis of the paw pads as those reported in the present case. Also similar to our findings, pyogranulomatous inflammation associated with fungal hyphae was identified on histopathology of the pericardium, ventricular myocardium and epicardium [4]. Interestingly, cardiac involvement has also been noted in approximately 50% of human cases with disseminated *Bipolaris* spp. infections [5]. The second canine case was reported in an 8-year old Labrador Retriever from Texas, presenting for polyuria and acute central vestibular signs. Pyogranulomatous lesions were identified in the kidneys and diffusely in the brain [6]. Treatment was not attempted in either case.

Other reported canine cases of phaeohyphomycosis have been caused by infections with *Phialemonium obovatum* [7], *Cladophialophora bantiana* [8], [9], [10], *Curvularia spp* [11], [12]*, Ochroconis gallopavum* [13], and *Phialemonium curvatum* [14]. Of these dogs, the majority died or were euthanized without antifungal treatment. Treatment for cerebral *C. bantiana* with fluconazole alone was unsuccessful [9], but surgical debulking of a cerebral *C. bantiana* granuloma, followed by fluconazole and later voriconazole successfully resulted in clinical remission 16 months after surgery [10]. Generalized cutaneous phaeohyphomycosis caused by *Curvularia lunata* has also been successfully treated with amphotericin B and itraconazole [12]. As in people, the prognosis for canine phaeohyphomycosis is guarded to poor, but immunocompetency, localized and surgically resectable disease, and treatment with the newer triazole compounds, voriconazole and posaconazole, may result in improved clinical outcomes.

A comprehensive review of case reports describing disseminated phaeohyphomycosis identified a total of 72 human cases from 1966 to 2002 [5]. *Bipolaris* organisms, including *B. spicifera, B. australiensis* and one unspeciated isolate accounted for 11% of cases. The prognosis for disseminated phaeohyphomycosis was exceptionally poor, with an overall mortality rate of 79%. A variety of treatments were attempted in this case compilation, including amphotericin B, ketoconazole, fluconazole, itraconazole, flucytosine, and granulocyte colony stimulating factor. No single treatment or combination of treatments was associated with improved survival. Given the sporadic nature of phaeohyphomycosis, randomized clinical trials are not feasible and reporting of clinical outcomes in individual cases is encouraged [2].

Similar to our findings with posaconazole, *in vitro* susceptibility testing of other *Bipolaris* spp. isolates supports its efficacy [1], [2]. There are also reports of successful treatment of disseminated phaeohyphomycosis in humans with voriconazole or posaconazole, alone or in combination with amphotericin B and echinocandins [15]. Fluconazole was used initially in this case due to concerns regarding the cost of other antifungal therapies and uncertainty regarding the meaning of a positive *Bipolaris* culture with histopathological lesions that initially appeared consistent with an immune-mediated process. A recent review of phaeohyphomycosis in a tertiary cancer center found that only 11% of 348 cultured isolates were associated with proven or probable disease [16]. However, given the poor susceptibility of *Bipolaris* spp. to fluconazole and the gravity of this infection, early treatment with itraconazole, voriconazole or posaconazole may have resulted in an improved outcome for this patient, despite rapid discontinuation of cyclosporine.

Treatment with cyclosporine likely contributed to the development of phaeohyphomycosis in this case. The trough concentration of 1731.4 ng/mL far exceeds the recommended trough concentrations of 400–600 ng/mL. Intestinal P-glycoprotein activity has been shown to influence absorption of cyclosporine in humans, but the same effect has not been demonstrated in dogs. Furthermore, this dog tested negative for the ABCD1 gene mutation commonly affecting P-glycoprotein expression in Miniature Australian Shepherds (Veterinary Clinical Pharmacology Lab, Washington State University, Pullman, WA), making excessive intestinal absorption an unlikely cause for her supra-therapeutic drug levels. Inhibition of cyclosporine metabolism by omeprazole via the CYP 3 A enzyme subfamily may have played a role in this case, although the effects of co-administration of omeprazole and cyclosporine are not consistent in all studies [17], [18]. Given the known variability in pharmacokinetics and potential for multiple drug interactions, this case highlights the importance of therapeutic drug monitoring of cyclosporine.

The culture of *C. glabrata* and *F. oxysporum* from multiple tissues at the time of necropsy is also significant, and, in the absence of any histopathologic evidence of organ infiltration, likely represents a terminal fungal sepsis in a severely immunocompromised patient. *Candida* spp., including *C. glabrata*, are normal commensal organisms of the upper gastrointestinal, respiratory, and lower urogenital tracts [19]. *F. oxysporum* is a soil saprophyte and plant pathogen, and is known to cause superficial cutaneous and nail bed infections in people [20]. Both can be responsible for life-threatening disseminated disease in an immunocompromised patient, and likely also played a role in this case.

## Conflict of interest

The authors report no conflicts of interest. The authors alone are responsible for the content and writing of the paper.
